# Development, optimization and integrated characterization of rice-based yogurt alternatives enriched with roasted and non-roasted sprouted barley flour

**DOI:** 10.1016/j.crfs.2025.101059

**Published:** 2025-04-24

**Authors:** Mario Caponio, Michela Verni, Ali Zein Alabiden Tlais, Edoardo Longo, Erica Pontonio, Raffaella Di Cagno, Carlo Giuseppe Rizzello

**Affiliations:** a Department of Soil, Plant, and Food Sciences, University of Bari, 70125, Bari, Italy; b Department of Environmental Biology, “Sapienza” University of Rome, 00185, Rome, Italy; c Faculty of Agricultural, Environmental and Food Sciences, Free University of Bozen-Bolzano, 39100, Bolzano, Italy; d International Center on Food Fermentation (ICOFF), NOI Tech Park, Via Ipazia 2, 39100, Bozen-Bolzano, Italy

**Keywords:** Sprouted barley, Germination, Yogurt alternatives, Fermentation, Viscosity, GABA

## Abstract

Plant-based yogurt substitutes (“gurts”), whose market growth is steadily increasing, have emerged as a promising option to promote more sustainable diets and food systems, especially when produced with locally sourced or low-input crops like barley. In this study, a novel gurt made with rice (10 %) and sprouted barley (5 %), was designed. Four lactic acid bacteria strains, *Levilactobacillus brevis* AM7, *Leuconostoc pseudomesenteroides* DSM20193, *Lactiplantibacillus plantarum* 18S9 and H64, were used as starters for making prototypes. Although with some differences in their acidification kinetics and proteolysis, all the strains adapted to the matrix. Then the formulation and production process were optimized. The use of sprouted barley, compared to raw flours, provided a content of amino acids 9-fold higher, further increased (up to 35 %) by the fermentation, and a more complex aroma profile characterized by the presence of furans and aldehydes. However, the high amylolytic activity in sprouted barley interfered with starch gelatinization decreasing the viscosity of the products from 3.3 to 0.08 Pa∗s. To overcome this challenge and obtain a creamy and spoonable product, sprouted barley flour was roasted, deactivating the enzymes and conferring a nutty and toasted flavor to the gurts due to the presence of pyrazines. The stability of the key biochemical and microbiological parameters during refrigerated storage was also assessed. Hence, plant-based gurts made with sprouted barley, emerge as a sustainable and health-promoting substitute to traditional dairy yogurts.

## Highlights

• Novel plant-based yogurt substitutes (“gurts”) with sprouted barley were designed.
• Amylolytic activity in sprouted barley interfered with starch gelatinization.
• Roasting of barley deactivated enzymes and conferred nutty and toasted notes.
• Fermentation with *Lactiplantibacillus plantarum* functionalized the gurts with GABA.
• Sprouted barley-gurt is a sustainable and health-promoting substitute to dairy yogurts.

## Graphical abstract

**Figure:**
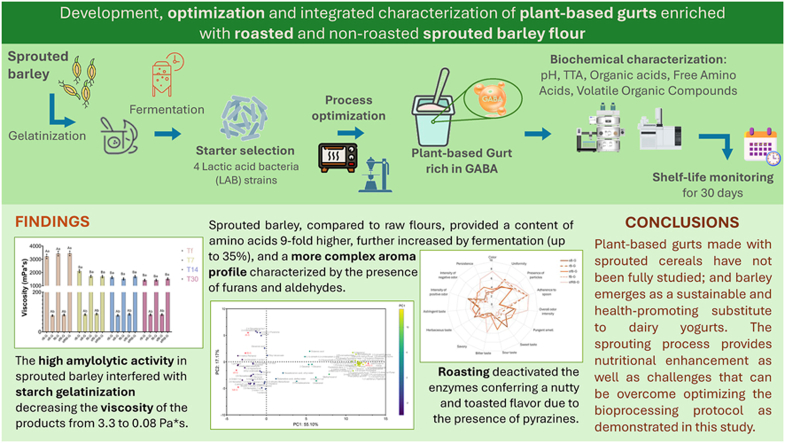

## 1 Introduction

The growing global awareness of the health, environmental, and ethical issues associated with animal-based diets led to a significant interest in plant-based alternatives. Among them, plant-based yogurts sometimes referred to as “gurts” (Demarinis et al., 2024; Montemurro et al., 2024; Wang et al., 2024) have emerged as a promising option to promote more sustainable diets (Medici et al., 2023). Indeed, the worldwide revenue in the yogurt substitutes market amounted to US $2.36 billion in 2024, with the market expected to grow annually by 6 % in the next five years (Statista, 2024). Plant-based yogurt alternatives are suitable not only to consumers with dairy allergies or ethical concerns but also to those seeking functional foods enriched with dietary fibres, bioactive compounds, and probiotics (Sethi et al., 2016).

Nevertheless, despite plant-based yogurts could potentially meet these characteristics, their production still faces challenges that need to be addressed to facilitate their acceptance and marketability. Such challenges are associated with three key areas: *i*) enhancing their organoleptic properties, to render appearance, texture, and flavour similar to the dairy counterpart, *ii*) improving the protein content and quality to match that of dairy-based yogurts, and *iii*) ensuring product stability during storage (Medici et al., 2023; Grasso et al., 2020). For these reasons, once the main ingredients are selected, a series of physical and biotechnological treatments are applied to obtain the physicochemical and sensory properties of traditional yogurts (Montemurro et al., 2021). Cereals such as oats, rice, wheat, and barley, are among the most commonly utilized ingredients in the formulation of gurts. They provide carbohydrates, dietary fibre, and proteins (Montemurro et al., 2021), and may also contain prebiotic compounds, as in the case of barley β-glucans.

Barley is a widely cultivated cereal crop that requires relatively low inputs of water, fertilizers, and pesticides, thereby making it a sustainable option for plant-based food production. Its resilience to harsh climates and ability to grow in marginal soils contribute to its ecological advantages (Kaur et al., 2024; Kumar et al., 2020). Therefore, the incorporation of barley flour in yogurt alternatives has the potential to diminish their environmental impact and promote sustainable dietary patterns. The use of raw cereal flours in these products has been well-documented, however, studies on plant-based gurts containing germinated flours are lacking. The germination process entails the controlled sprouting of grains, during which endogenous enzymes that break down complex molecules into simpler forms are activated (Marti et al., 2020). Compared to the use of raw flours, the incorporation of sprouted flours to the gurts, in conjunction with fermentation, could result in an enhanced bioavailability of bioactive compounds (e.g., peptides, amino acids, phenolic compounds) and a more complex organoleptic profile. Moreover, the germination process boosts the levels of certain vitamins, particularly those of the B-group such as riboflavin and niacin; increases soluble dietary fiber, which can improve digestive health; and reduces anti-nutritional compounds like phytic acid thus enhancing the bioavailability of minerals such as zinc, iron, magnesium, and phosphorus (Marti et al., 2020; Ikram et al., 2021; Elliott et al., 2022; Lemmens et al., 2019).

Therefore, based on the above considerations, this paper focused on the optimization of a lactose-free gurt containing sprouted barley flour. The design included the selection of the lactic acid bacteria (LAB) used as starters, the optimization of the production process, and the characterization of the gurt after fermentation and during refrigerated storage.

## 2 Materials and methods

### 2.1 Raw materials and microorganisms

The ingredients used in this study include rice flour (Molino Peila SPA, Valperga, Italy) and barley grains (*Hordeum vulgare* L.) purchased from an Apulian local market.

*Lactiplantibacillus plantarum* 18S9, isolated from hemp (Nionelli et al., 2018a), *Lactiplantibacillus plantarum* H64 isolated from hop (Nionelli et al., 2018b), *Levilactobacillus brevis* AM7, previously used for the fermentation of plant-based gurts (Demarinis et al., 2024), and *Leuconostoc pseudomesenteroides* DSM20193 (Koirala et al., 2021) were used as starters. All strains were cultivated at 30 °C in De Man, Rogosa and Sharpe (MRS) Broth (Oxoid, Basingstoke, Hampshire, United Kingdom) until the late exponential phase of growth was reached (ca. 16 h), then cells were harvested by centrifugation at 10,000×*g* for 10 min at 4 °C; washed twice in sterile 50 mM phosphate buffer, pH 7.0, and resuspended in tap water before use.

### 2.2 Sprouting process and flour production

Barley kernels were germinated following the procedure previously described by Perri et al. (2023). Briefly, barley was submerged in 1.25 % sodium hypochlorite (NaClO) at room temperature for 30 min. Then, grains, washed under running water for other 30 min to remove sodium hypochlorite residues, were soaked for 24 h in tap water (seed:water ratio of 1:5). Kernels were placed in a household germinator (Whirlpool, Benton Harbor, MI, USA) in the dark at 20 °C and sprinkled with tap water every 12 h. Germination was stopped when rootlets reached about 3/4 of the seed's length (ca. 3 days). Then germinated kernels were thoroughly washed with distilled water and dried for 24 h at 50 °C in a professional oven (VEVOR EB-4D, VEVOR, Germany). The drying conditions are comparable to those used for industrial malting of barley and suitable for the preservation of enzymatic activity (Laitila et al., 2006). After drying, the seeds were milled with a laboratory mill (IKA A11 basis, Werke, Germany).

### 2.3 Starter selection

For the gurt making, rice and sprouted barley flours were resuspended in tap water at 15 and 5 %, respectively, and subjected to a gelatinization process at 80 °C for 15 min. Then, the gelatinized mixture (100 mL for each replicate) was cooled at 30 °C prior to the inoculum of the starters. Strains were singly inoculated at a cell density of approx. 7.0 log cfu/g. Fermentation was carried out at 30 °C for 18 h. The pH was monitored with a FiveEasy FP20 pHmeter (Mettler Toledo, USA), equipped with a with a food penetration probe, at 1-h intervals during the first 12 h, and then at 16, and 18 h. Data were modelled according to the Gompertz equation, modified by Zwietering et al. (1990):

$$y=k+A\;\exp\left\{-\exp\left[\left(V_{\max}\;e/A\right)\left(-t\right)+1\right]\right\}$$

where y is the acidification rate expressed as ΔpH/Δt (unit of pH/h); k is the initial level of the dependent variable to be modelled; A is the difference in pH (ΔpH) between the initial value of pH and that reached at the stationary phase; V_max_ is the maximum rate of acidification expressed as ΔpH/h; λ is the length of the adaptation phase measured in hours.

At the end of fermentation, total titratable acidity (TTA) was measured on 10 g of sample diluted with 90 mL of distilled water and expressed as the amount (mL) of 0.1 M NaOH necessary to reach a pH 8.3. LAB were enumerated using MRS Agar (Oxoid) supplemented with cycloheximide (0.1 g/L). Plates were incubated, under anaerobiosis (AnaeroGen and AnaeroJar, Oxoid) at 30 °C for 48 h.

Water-salt soluble extracts (WSE) were obtained following the method initially described by Osborne and modified by Weiss (Weiss et al., 1993) using 50 mM Tris-HCl (pH 8.8). The suspension was kept at 4 °C for an hour, vortexed every 15 min and, centrifuged at 12,000×*g* for 20 min. The supernatant was collected in new falcon. The total free amino acid (TFAA) content was determined by the cadmium-ninhydrin assay as described in Verni (Verni et al., 2024).

The viscosity was measured by a rotational viscometer Lichen NDJ-8S (LICHEN, Zhejiang, China). The measurements were performed before and after fermentation at 25 °C, in a 7 × 12.5 cm container, considering different probes and rotation speeds.

### 2.4 Optimization of the gurt production process

Aiming at overcoming the technological issues emerged during the starter selection, the production process of the beverage was optimized, and five new gurts were produced, using rice flour (15 %) and raw or sprouted barley flours (5 %) gelatinized as described above (Table 1). Two not inoculated controls were produced using raw (rB-G) or sprouted (sB-G) barley flours, chemically acidified with lactic acid (to a pH of 4.5) and incubated under the same conditions. Two more samples were inoculated with *L. plantarum* H64 (initial cell density of ca. 7.0 log cfu/g) and fermented at 30 °C for 8 h (fB-G and sfB-G). Another gurt (sfRB-G) was produced using sprouted barley treated at 150 °C for 3 h, to inactivate the enzymes overexpressed during germination, and fermented in the same condition as the others.

**Table 1:** List of sample codes and ingredients contained in each gurt.

Code	Ingredients
rB-G	Rice and raw barley flours (chemically acidified control)
sB-G	Rice and sprouted barley flours (chemically acidified control)
fB-G	Rice and barley flours (fermented with *L. plantarum* H64)
sfB-G	Rice and sprouted barley flours (fermented with *L. plantarum* H64)
sfRB-G	Rice and roasted sprouted barley flours (fermented with *L. plantarum* H64)

### 2.5 Characterization of the gurts

#### 2.5.1 Acidification, microbial stability and viscosity

Presumptive LAB were enumerated on MRS as described above. The pH of the samples was monitored during fermentation at 1-h intervals, to determine the kinetic of acidification, and TTA was also evaluated as described in paragraph 2.3. The determination of lactic and acetic acids was carried out by using the Megazyme K-DLATE and K-ACETRM (Megazyme International Ireland) kits, respectively, following the manufacturer's instructions. Glucose, maltose, and sucrose were analyzed with the K-MASUG (Megazyme) kit. The viscosity of the gurts was carried out using a rotational viscosimeter as described above.

#### 2.5.2 Analysis of free amino acids and volatile organic compounds (VOC)

Free amino acids and γ-amino butyric acid (GABA) were determined on WSE using the Biochrom 30+ Amino Acid Analyzer (Biochrom Ltd, Cambridge Science Park, UK) with a Li cation exchange column (20 × 0.46 cm internal diameter) (Verni et al., 2024).

For the analysis of VOC, each sample was prepared by weighing 0.2 g sodium chloride and 2 g of beverage inside 10-mL SPME glass vial sealed with a magnetic screw cap with polytetrafluoroethylene (PTFE)/silicone septa. 3 μL of internal standard (4-methyl-2-pentanol in ethanol, 1000 mg/L) were added to the samples before sealing. The SPME was performed with an LPAL3 GC autosampler equipped with a Peltier Stack, where the samples were kept at 4 °C before the analysis. Firstly, the samples were subjected to a 30-min conditioning at 40 °C. Then a pre-conditioned triphasic SPME fiber (50/30 μm DVB/CAR/PDMS, Stableflex, 23 Ga, 1 cm—Sigma-Aldrich, St. Louis, MO, USA) was inserted into the sample headspace. The GC/MS analysis was performed on a GC coupled to a Pegasus BT 4D time-of-flight mass spectrometer and equipped with a FluxTM flow modulator (Leco Italy, Milano). The separation was performed at 1 mL/min (He carrier gas) in split-less mode, with 2 mL/min septum purge flow and 6 mL/min inlet purge flow. The inlet temperature was 240 °C. Each sample was analyzed on a polar cross-bond PEG-phase MEGA-Wax Spirit 0.30 μm × 0.18 mm × 40 m (Mega S.r.l., Legnano, Italy). The temperature ramp of the main GC oven was 40 °C for 6 min (at injection), then ramped from 40 °C to 230 °C at 3 °C/min. The mass spectrometer operated in EI mode at 70 eV. The mass range was 34–360 m/z at 1 spectrum·s ^−1^; the temperature of the ion source and quadrupole were 230 °C and 150 °C, respectively. Replicates were strictly analyzed in a randomized mode. GC/MS data integration was performed automatically using the provided software (ChromaToF, LECO). Peaks were expressed as areas (for extracted ion current, XIC) vs. retention time and were aligned manually. The compounds were assigned to chemical species by comparison of the acquired spectra with reference mass spectra (NIST, 2011 database) and by calculating the linear retention indexes (LRI). The linear retention indexes were calculated against linear saturated alkanes.

#### 2.5.3 Sensory analysis

Sensory analysis of the gurts was performed by a trained panel group composed of ten assessors (5 male and 5 females, mean age: 32 years, range: 24–46 years) with proven skills and previous experiences in sensory evaluation of cereal-based products. The sensory attributes, scored on a scale from 0 to 10 (with 10 the highest score), were discussed with the assessors during the introductory 2 h-training session. Sensory evaluations were carried out following the independent method of the “Sensory analysis - Methodology - Flavour Profile” methods (ISO 6564–1985) with some modification. As previously proposed by Elia (2011), the library of the Environmental Biology Department of the Sapienza University of Rome (Italy) was used instead of cabinets. Gurts were served in a randomized order after cooling at 4 °C for 3 h. A glass of water was drunk by the panellists after each sample analysis.

#### 2.5.4 Shelf-life

Aiming at evaluating the chemical, textural, and microbiological stability of the gurts during the shelf-life, after 7 (T7), 14 (T14), and 30 (T30) days of refrigerated storage at 4 °C, the main biochemical parameters (pH, TTA, organic acids, sugars, and TFAA) as well as LAB survival and viscosity were determined as described above.

### 2.6 Statistical analysis

The analyses were carried out on samples obtained in three separate replicates and each sample was analyzed in duplicate. Data of the starter selection were subjected to one-way ANOVA; paired comparison of treatment means was achieved by Tukey's procedure at P*<*0.05, using the software Statistica 12.5 (StatSoft Inc., Tulsa, USA). Data of the optimization process were subjected to two-way ANOVA; the significance of differences (P < 0.05) between mean values was evaluated by Tukey's procedure as described above. Data obtained from the analysis of VOC were subjected to Principal Component Analysis (PCA) using GraphPad Prism 7.0 (GraphPad Software, Inc., San Diego, CA).

## 3 Results

### 3.1 Starter selection

**Fig. 1:**
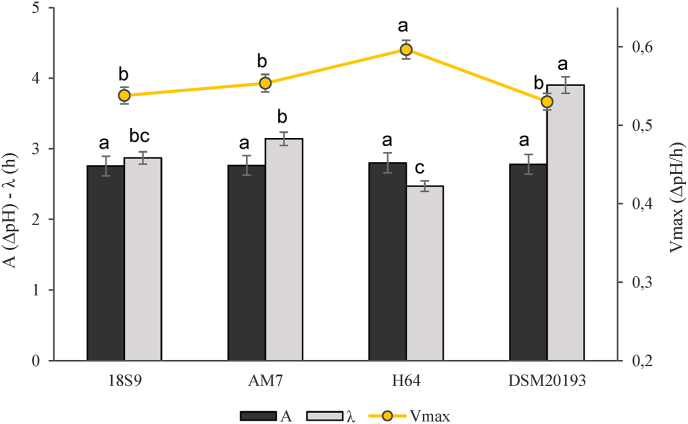
Acidification kinetics parameters of the gurts containing rice and sprouted barley flours, fermented with *L. platarum* 18S9 (18S9), *L. brevis* AM7 (AM7), *L. plantarum* H64 (H64), and *Leuc. mesenteroides* DSM20193 (DSM20193). A, ΔpH between the initial value of pH and that reached at the stationary phase; V_max_, maximum rate of acidification expressed as ΔpH/h; λ, length of the adaptation phase measured in hours. The data are the means of three independent experiments ± standard deviation. ^a-c^Values with different superscript letters differ significantly (P < 0.05).

Aiming at selecting the most suitable starter for fermentation of the rice and sprouted barley matrix, a preliminary characterization based on their pro-technological properties was performed. Cell densities of the strains increased of ca. 2 log cycles, reaching 9 log cfu/g. Each strain was able to ferment and acidify the matrix. On averages values of pH and TTA of 3.35 and 8.5 mL were reached, respectively, with no significant differences (P > 0.05) among the strains. However, by monitoring the acidification of gurts through the Gompertz equation, differences between strains were observed (Fig. 1). Specifically, *Leuc.* *pseudomesenteroides* DSM20193 and *L. brevis* AM7 had respectively the lowest acidification rate (0.53 ΔpH/h) and the highest lag phase (3.4 h).

Before fermentation, TFAA were 254 mg/kg, yet during fermentation, in all matrices, except for those fermented with *L. plantarum* H64, a decrease between 10 % and 20 % was observed.

The viscosity of the matrices did not increase during the gelatinization and remained below 100 mPas∗s for all strains. Based on these results, an optimization of the production process was conducted considering only *L. plantarum* H64 which showed a good capacity of growth and acidification and did not lead to a decrease in total free amino acids, compared to the other strains.

### 3.2 Optimization of production process

#### 3.2.1 Growth and acidification

The optimization involved the modification of the fermentation time from 18 to 8 h, necessary to reduce the acidification. Hence four new gurts were obtained using raw or sprouted barley flour, two of them were fermented with *L. plantarum* H64 (fB-G and sfB-G) and two controls were chemically acidified to exclude the effect of pH on the viscosity (rB-G and sB-G). A fifth sample was produced with roasted sprouted barley (sfRB-G).

During fermentation, even if short, *L. plantarum* grew more than 1 log cycle reaching cell densities of 8.7 log cfu/g in sfRB-G. In fB-G and sfB-G, at the end of fermentation, presumptive LAB exceeded 9 log cfu/g, whereas in not-inoculated controls (rB-G and sB-G), which were incubated in the same conditions, LAB remained below 2 log cfu/g.

**Fig. 2:**
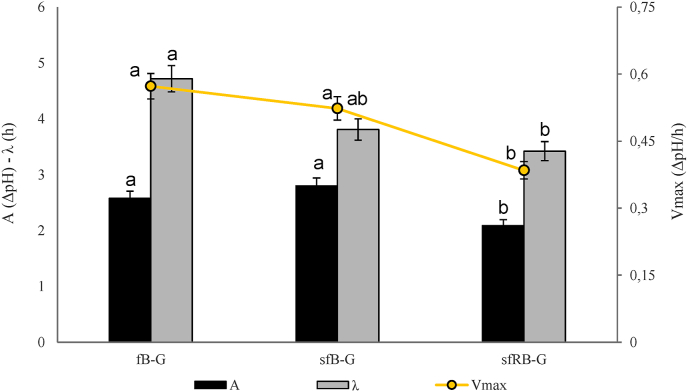
Acidification kinetics parameters of the barley-based gurts. rB-G and sB-G, not fermented controls respectively produced with raw or sprouted barley flours and chemically acidified with lactic acid; fB-G and sfB-G, gurts respectively produced with raw or sprouted barley flours and fermented with *L. plantarum* H64; sfRB-G, fermented gurt produced with roasted sprouted barley. A, ΔpH between the initial value of pH and that reached at the stationary phase; V_max_, maximum rate of acidification expressed as ΔpH/h; λ, length of the adaptation phase measured in hours. The data are the means of three independent experiments ± standard deviation. ^a-c^Values with different superscript letters differ significantly (P < 0.05).

The starter efficiently acidified all beverages reaching pH values from 3.91 to 4.68, respectively in sfB-G and fB-G. Among fermented samples, significant differences (P < 0.05) in the acidification kinetics were observed (Fig. 2). Although those containing sprouted barley had lower latency phase compared to fB-G, the influence of the heat treatment (in sfRB-G) resulted in clear decreases (P < 0.05) of both the maximum acidification rate (V_max_) and the pH variation between inoculum and stationary phase (A) compared to sfB-G.

Similarly, at the end of fermentation, TTA was higher in sfB-G (7.6 ± 0.4 mL) compared to the other samples (Table 2). No significant differences (P > 0.05) were found between chemically acidified controls, nor between fB-G and sfRB-G, with an average of 4 mL (Table 2). The higher TTA found especially in sfB-G, was consistent with the production of organic acids. Indeed, values of 14 and 1.5 mmol/kg of lactic and acetic acids, respectively, were found in sfB-G (Table 2).

**Table 2:** Main biochemical and microbiological characteristics of the gurts before (t0) and after incubation at 30 °C for 8 h (tf), and after 7 (t7), 14 (t14), and 30 (t30) days of storage at 4 °C. rB-G and sB-G, not fermented controls respectively produced with raw or sprouted barley flours and chemically acidified with lactic acid; fB-G and sfB-G, gurts respectively produced with raw or sprouted barley flours and fermented with *L. plantarum* H64; sfRB-G, fermented gurts produced with sprouted barley treated at 150 °C for 3 h.

	pH	TTA (mL)	LAB (log cfu/g)	Lactic Acid (mmol/kg)	Acetic Acid (mmol/kg)	Glucose (mmol/kg)	Maltose (mmol/kg)	Sucrose (mmol/kg)	TFAA (mg/kg)
**t0**									
**rB-G**	4.20 ± 0.02^Ac^	4.4 ± 0.0^Ca^	1.54 ± 0.07^Bc^	9.58 ± 0.22^Da^	n.d.	24.2 ± 0.35^Ab^	11.3 ± 0.64^Ab^	2.2 ± 0.49^Bb^	27.2 ± 1.3^Ac^
**sB-G**	4.17 ± 0.01^Ac^	4.9 ± 0.1^Ca^	1.84 ± 0.04^Bb^	8.99 ± 0.19^Da^	n.d.	50.1 ± 0.92^Ca^	22.5 ± 0.99^Aa^	8.2 ± 0.42^Aa^	252 ± 17^Aa^
**fB-G**	5.94 ± 0.01^Aa^	0.4 ± 0.0^Dc^	7.26 ± 0.02^Ba^	n.d.	n.d.	21.3 ± 0.35^Cb^	12.8 ± 1.41^Ab^	2.0 ± 0.84^Bb^	31.2 ± 2.3^Cc^
**sfB-G**	5.96 ± 0.03^Aa^	1.8 ± 0.0^Cb^	7.16 ± 0.06^Ba^	n.d.	n.d.	46.3 ± 1.34^Ca^	25.1 ± 0.85^Aa^	8.1 ± 0.71^Aa^	249 ± 13^Ca^
**sfRB-G**	5.57 ± 0.02^Ab^	1.5 ± 0.1^Eb^	7.25 ± 0.03^Da^	n.d.	n.d.	5.5 ± 0.07^Cc^	0.3 ± 0.10^Ac^	3.3 ± 0.21^Ac^	122.5 ± 9.4^Cb^
**tf**									
**rB-G**	4.22 ± 0.03^Ab^	4.4 ± 0.2^Cb^	1.61 ± 0.07^Bc^	9.47 ± 0.25^Dbc^	n.d.	25.0 ± 1.75^Ac^	6.9 ± 0.28^Ba^	4.0 ± 0.25^Ab^	28.0 ± 2.2^Ae^
**sB-G**	4.20 ± 0.04^Ab^	5.1 ± 0.1^Cb^	1.79 ± 0.04^Bc^	8.93 ± 0.25^Dc^	n.d.	99.5 ± 1.98^Ba^	0.2 ± 0.05^Bc^	2.5 ± 0.84^Bc^	252 ± 16^Ab^
**fB-G**	4.68 ± 0.05^Ba^	3.8 ± 0.1^Cc^	9.01 ± 0.10^Aa^	9.43 ± 0.06^Cb^	0.70 ± 0.10^Cb^	48.1 ± 0.14^Ab^	6.9 ± 0.64^Ba^	5.0 ± 0.13^Aa^	81.2 ± 5.4^Bd^
**sfB-G**	3.91 ± 0.05^Bc^	7.6 ± 0.4^Ba^	9.12 ± 0.11^Aa^	14.00 ± 0.10^Ca^	1.47 ± 0.06^Ca^	91.5 ± 1.56^Aa^	4.4 ± 0.27^Bb^	1.5 ± 0.05^Bc^	339 ± 22^Bba^
**sfRB-G**	4.70 ± 0.09^Ba^	3.9 ± 0.2^Dc^	8.68 ± 0.14^Cb^	6.10 ± 0.00^Cd^	1.00 ± 0.10^Cab^	13.5 ± 0.99^Ad^	0.4 ± 0.07^Ac^	4.0 ± 0.57^Ab^	162 ± 12^Bc^
**t7**									
**rB-G**	4.06 ± 0.03^Bb^	6.1 ± 0.1^Bd^	1.73 ± 0.02^Bd^	10.77 ± 0.15^Cd^	n.d.	24.8 ± 0.98^Ac^	6.3 ± 0.32^Ba^	3.8 ± 0.17^Aa^	30.3 ± 2.9^Ae^
**sB-G**	4.26 ± 0.01^Aa^	7.8 ± 0.2^Bc^	1.89 ± 0.01^Bc^	9.30 ± 0.00^Cd^	n.d.	101.5 ± 0.76^ABa^	0.1 ± 0.29^Bc^	2.1 ± 0.04^Bb^	261 ± 15^Ab^
**fB-G**	4.11 ± 0.02^Cb^	9.6 ± 0.1^Bb^	9.20 ± 0.09^Aa^	12.90 ± 0.20^Bb^	1.07 ± 0.06^Bc^	47.6 ± 0.28^Ab^	6.0 ± 0.37^Ba^	4.2 ± 0.24^Aa^	97.2 ± 7.1^ABd^
**sfB-G**	3.53 ± 0.02^Cc^	11.8 ± 0.2^Aa^	9.22 ± 0.07^Aa^	14.83 ± 0.15^Ba^	1.77 ± 0.06^CBb^	98.7 ± 0.97^Aa^	4.0 ± 0.05^Bb^	1.1 ± 0.07^Bc^	365 ± 25^ABa^
**sfRB-G**	4.18 ± 0.01^Cab^	6.4 ± 0.0^Cd^	8.99 ± 0.01^Bb^	10.50 ± 0.20^Bc^	2.20 ± 0.20^Ba^	12.6 ± 0.07^Ad^	0.1 ± 0.02^Bc^	3.7 ± 0.57^Aa^	198 ± 14^ABbc^
**t14**									
**rB-G**	4.15 ± 0.01^Ba^	6.7 ± 0.1^Bc^	1.82 ± 0.03^Bb^	11.40 ± 0.00^Bc^	n.d.	19.9 ± 1.27^Ad^	5.9 ± 0.13^Ba^	2.9 ± 0.59^Ba^	32.9 ± 2.1^Ad^
**sB-G**	4.25 ± 0.02^Aa^	7.0 ± 0.3^Bc^	1.94 ± 0.07^Bb^	10.10 ± 0.10^ABd^	n.d.	106.9 ± 0.84^Aa^	0.0 ± 0.01^Bc^	1.8 ± 0.41^Bb^	260 ± 13^Ab^
**fB-G**	3.80 ± 0.02^Db^	9.9 ± 0.0^Bb^	9.23 ± 0.13^Aa^	15.00 ± 0.00^Aa^	2.10 ± 0.10^Ab^	44.2 ± 0.81^Bc^	5.3 ± 0.21^BCa^	2.3 ± 0.18^Ba^	112±8^Ac^
**sfB-G**	3.50 ± 0.05^Cc^	12.1 ± 0.2^Aa^	9.28 ± 0.24^Aa^	15.07 ± 0.06^ABa^	3.10 ± 0.10^Aa^	96.3 ± 0.77^Bb^	3.9 ± 0.17^Bb^	0.8 ± 0.13^Bc^	393 ± 32^Aa^
**sfRB-G**	3.77 ± 0.03^Dd^	7.7 ± 0.00^Bc^	9.10 ± 0.02^Aa^	14.23 ± 0.21^Ab^	2.83 ± 0.15^Aa^	11.9 ± 0.29^Be^	0.2 ± 0.03^Bc^	2.1 ± 0.18^Bab^	232 ± 26^Ab^
**t30**									
**rB-G**	4.01 ± 0.01^Cb^	8.4 ± 0.00^Ac^	2.18 ± 0.07^Ac^	12.20 ± 0.17^Ab^	n.d.	20.3 ± 1.41^Ad^	5.7 ± 0.07^Ba^	2.6 ± 0.17^Ba^	30.5 ± 2.8^Ad^
**sB-G**	4.32 ± 0.02^Aa^	8.8 ± 0.06^Ac^	2.65 ± 0.08^Ab^	10.67 ± 0.23^Ac^	n.d.	105.1 ± 0.99^Aa^	0.1 ± 0.03^Bc^	1.6 ± 0.27^Bb^	268 ± 18^Ab^
**fB-G**	3.76 ± 0.00^Dc^	10.8 ± 0.00^Aa^	9.36 ± 0.14^Aa^	14.83 ± 0.15^Aa^	1.63 ± 0.20^Ab^	43.7 ± 0.11^Bc^	5.2 ± 0.14^Ca^	1.9 ± 0.22^Bb^	120±7^Ac^
**sfB-G**	3.38 ± 0.03^Dd^	11.2 ± 0.10^Aa^	9.47 ± 0.15^Aa^	15.67 ± 0.23^Aa^	2.33 ± 0.15^ABb^	94.9 ± 0.68^Bb^	4.2 ± 0.24^Bb^	0.9 ± 0.09^Bc^	398 ± 25^Aa^
**sfRB-G**	3.35 ± 0.00^Ed^	9.2 ± 0.00^Ab^	9.24 ± 0.17^Aa^	14.33 ± 0.55^Aa^	3.93 ± 0.21^Aa^	12.3 ± 0.18^ABe^	0.1 ± 0.05^Bc^	1.9 ± 0.23^Bb^	239 ± 23^Ab^

TTA, Total Titratable Acidity; LAB, Lactic Acid Bacteria; TFAA, Total Free Amino Acids.

The data are the means of three independent experiments ± standard deviation.

Values in the same column with different uppercase letters, among different incubation times of the same sample, mean significant differences at a P < 0.05. Different lowercase letters, among different samples within the same incubation time, mean significant differences at a P < 0.05.n.d., not detected.

#### 3.2.2 Biochemical characterization

**Fig. 3:**
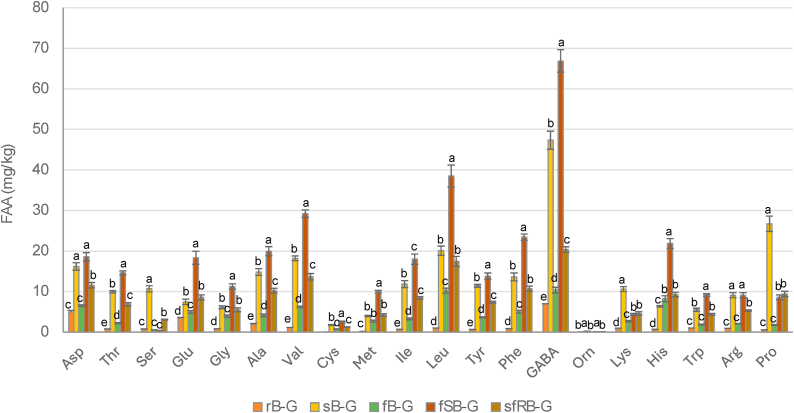
Free amino acids concentration (mg/kg) of the barley-based gurts. rB-G and sB-G, not fermented controls respectively produced with raw or sprouted barley flours and chemically acidified with lactic acid; fB-G and sfB-G, gurts respectively produced with raw or sprouted barley flours and fermented with *L. plantarum* H64; sfRB-G, fermented gurt produced with roasted sprouted barley. The data are the means of three independent experiments ± standard deviation. ^a-e^Values with different superscript letters, within the same amino acid, differ significantly (P < 0.05).

All the gurts were characterized for the main biochemical features, including sugars, free amino acids, and volatile organic compounds. Evidently, the two controls had a different content of glucose, maltose and sucrose, which was at least doubled in sB-G compared to rB-G (Table 2). Overall, a general increase of glucose and decrease of maltose was observed after the incubation, especially in samples containing sprouted barley. The two not-fermented controls differed for the TFAA content, which was almost 10-fold higher in sB-G compared to rB-G (Table 2). In rB-G, Asp and Glu were the most abundant amino acids whereas in sB-G, GABA, Leu, and Pro were found in the highest concentration. Except for sfRB-G, after fermentation, an increase (up to 190 %) of total and single amino acids was observed compared to their respective controls. Samples containing sprouted barley (sB-G and sfB-G) had the highest GABA content (47 and 67 mg/kg, respectively), however, the roasting resulted in a reduction (approx. 3 times) of GABA (Fig. 3).

**Fig. 4:**
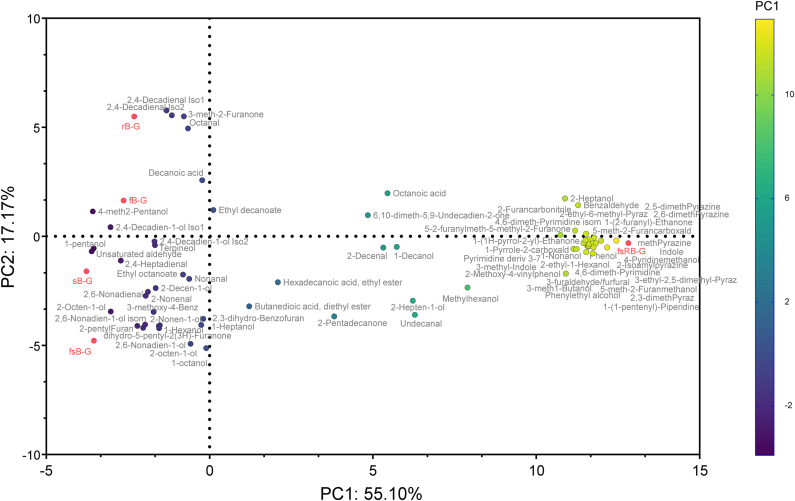
Principal component analysis based on the Volatile Organic Compounds profile of the barley-based gurts. rB-G and sB-G, not fermented controls respectively produced with raw or sprouted barley flours and chemically acidified with lactic acid; fB-G and sfB-G, gurts respectively produced with raw or sprouted barley flours and fermented with *L. plantarum* H64; sfRB-G, fermented gurt produced with roasted sprouted barley.

The analysis of the VOC in the gurts revealed a total of 74 identified compounds which included carboxylic acids, alcohols, esters, aldehydes, ketones, furans, aromatic heterocycles and heterocyclic compounds. The most abundant compound across all samples was 4-methyl-2-pentanol, followed by 1-hexanol. Whereas methylhexanol was only detected in fermented samples. The unsupervised PCA analyses revealed distinct clustering of gurts based on their volatile compound profiles (Fig. 4). Factor 2, representing 17.17 % of the total variance, clearly separated gurts obtained with raw and sprouted barley, where the latter were characterized by compounds like 2-pentylfuran and 3-methoxy-4-[(2-methylphenyl)methoxy]-benzaldehyde and the former by 3-meth1-butanol and 5-(2-furanylmethyl)-5-methyl-2(5H)-furanone each not present in the other group. Factor 1, instead, representing 55.10 % of the total variance, separated sfRB-G from the other samples. Indeed, sfRB-G was characterized by the presence of several pyrazine (methyl-pyrazine, 2,5-dimethyl-pyrazine, 2,3-dimethyl-pyrazine, 2-ethyl-6-methyl-pyrazine, 3-ethyl-2,5-dimethyl-pyrazine, 2-Isoamylpyrazine) and pyrimidine derivatives, which were not detected in any of the other samples (Fig. 4).

#### 3.2.3 Viscosity and sensory analysis

**Fig. 5:**
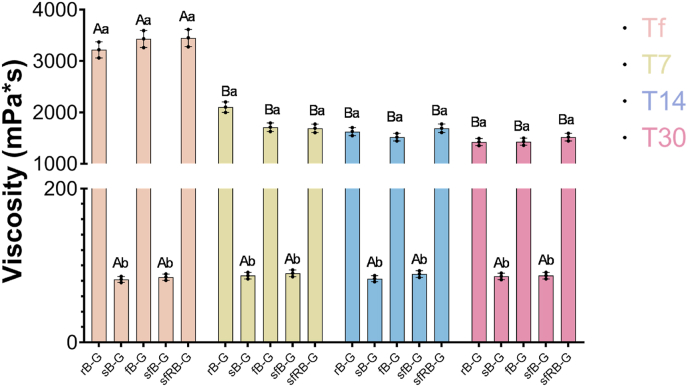
Viscosity, expressed as mPa∗s, of the gurts before (t0) and after (tf) fermentation and after 7 (t7), 14 (t14), and 30 (t30) days of refrigerated storage. rB-G and sB-G, not fermented controls respectively produced with raw or sprouted barley flours and chemically acidified with lactic acid; fB-G and sfB-G, gurts respectively produced with raw or sprouted barley flours and fermented with *L. plantarum* H64; sfRB-G, fermented gurt produced with roasted sprouted barley. The data are the means of three independent experiments ± standard deviation. Different uppercase letters between different incubation time of the same sample mean significant differences at a P < 0.05. Different lowercase letters among different samples within the same time of incubation mean significant differences at a P < 0.05.

Gurts containing raw barley (rB-G and fB-G) had a viscosity of roughly 3.3 Pa∗s, while those containing sprouted barley (sB-G and sfB-G) the viscosity was around 80 mPa∗s (Fig. 5). On the contrary, sfRB-G showed a viscosity comparable (3.45 ± 0.18 Pa∗s) to that of rB-G and fB-G (P > 0.05).

**Fig. 6:**
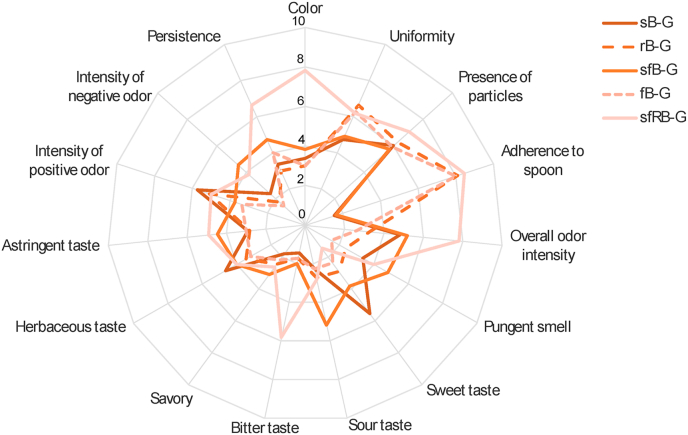
Sensory analysis of analysis of the barley-based gurts. rB-G and sB-G, not fermented controls respectively produced with raw or sprouted barley flours and chemically acidified with lactic acid; fB-G and sfB-G, gurts respectively produced with raw or sprouted barley flours and fermented with *L. plantarum* H64; sfRB-G, fermented gurt produced with roasted sprouted barley.

Aiming at describing the sensory profile of the gurts and highlighting the effect of the production process, in comparison to chemically acidified controls, samples were subjected to sensory analysis (Fig. 6). During the preliminary session, descriptors for the appearance, smell, taste, and aftertaste were selected. Overall, samples containing sprouted barley, either raw or fermented were characterized by a more intense aroma especially sfRB-G which also distinguished for the darker colour, almost hazelnut-like, compared to the other samples which were straw-coloured. Sprouted barley also conferred a sweeter taste to the gurts, which decreased and was covered by bitter and toasty notes in sfRB-G. As confirmed by the viscosity analysis, rB-G, fB-G and sfRB-G were characterized by a good adherence to the spoon, similar to conventional yogurt, whereas sB-G and sfB-G presented a more liquid texture.

### 3.3 Monitoring during storage

The main microbial, biochemical, and technological parameters were monitored during the refrigerated storage (Table 2, Fig. 5). LAB cell density remained stable around 9 log cfu/g, for fermented beverages whereas it was lower than 2 log cfu/g for non-fermented controls (rB-G and sB-G) up until the fourteenth day of storage and exceeded it in sB-G by the end of storage. Due to the increase of organic acids content, a gradual decrease of pH and increase of TTA were observed in all samples the first 2 weeks at 4 °C, then stabilized by the end of storage (Table 2). From the second week of storage, an increase in TFAA content, was observed in all fermented samples, reaching up to 400 mg/kg in sfB-G, whereas sugars, mostly sucrose and maltose slightly decreased in the last few weeks of storage.

As for the viscosity, even though a significant (P < 0.05) decrease was observed during storage of rB-G, fB-G and sfRB-G, compared to that obtained at the end of fermentation, the values remained in the same order of magnitude. Indeed, while the samples containing sprouted barley (sB-G and sfB-G) remained liquid with viscosity values lower than 0.1 Pa∗s, rB-G, fB-G, and sfRB-G showed higher viscosity with average values of 1.6 Pa∗s.

## 4 Discussion

Given their worldwide market potential (Statista, 2024), the replacement of conventional yogurts with plant-based alternatives has the potential to profoundly impact the sustainability of the food system. Conventional dairy production is frequently linked to substantial greenhouse gas emissions, land use, and water consumption (Carlsson Kanyama et al., 2021; Singaravadivelan et al., 2023). Conversely, plant-based gurts have been shown to have a comparatively reduced environmental impact, particularly when utilizing locally sourced or low-input crops like barley (Kaur et al., 2024; Kumar et al., 2020; Carlsson Kanyama et al., 2021). Moreover, these products align with dietary guidelines recommending a reduction in saturated fats and cholesterol intake (Medici et al., 2023). Cereal-based gurts can also contribute to a diversified food system by providing alternative protein sources and reducing reliance on soy or almond-based products, which have their own environmental concerns and are considered to have higher emissions than milk (Carlsson Kanyama et al., 2021). By incorporating barley and other selected cereals, plant-based gurts could address both nutritional and ecological challenges, offering a compelling solution for modern consumers. Nevertheless, the design of gurts with desirable texture and sensory properties has been challenging, and a multitude of aspects, from the selection of the right formulation to the bioprocesses necessary to impart the appropriate structure, mitigate sensory defects or reduce antinutritional factors need to be further explored (Dhakal et al., 2023). Whereas processes such as gelatinization, homogenization, LAB fermentation, supplement addition appear to be indispensable steps in the production of gurts (Montemurro et al., 2021; Dhakal et al., 2023), so far, very few studies have explored the possibility of using sprouted flours (Cáceres et al., 2019; Hwang et al., 2018; Ujiroghene et al., 2019), therefore including the germination of the grains. Indeed, it was observed that the combination of germination and fermentation can be used to increase the amount of bioactive compounds like GABA, phenolic compounds and peptides with antioxidant and antihypertensive activity of soy, quinoa or brown rice derived yogurt alternatives (Cáceres et al., 2019; Hwang et al., 2018; Ujiroghene et al., 2019; Zhang et al., 2023), thus effectively improving the overall nutritional and functional quality of such products.

### 4.1 Starter selection

Hence, based on the above considerations, this study aimed at designing a cereal gurt containing sprouted barley. Four LAB strains were used as starters for evaluating yogurt alternative prototypes. Two of them were able to synthesise exopolysaccharides, namely, due to its high dextran-producing ability *Leuc.* *pseudomesenteroides* DSM20193 is often used for the fermentation of cereal- and legume-based matrices including gurts (Koirala et al., 2021; Demarinis et al., 2023; Galli et al., 2021; Xu et al., 2018). Whereas *Lv.* *brevis* AM7 heteroexopolysaccharide was only recently characterized and its effect on the techno-functional properties of oat or carob and chickpea yogurt type was studied (Wang et al., 2024; Demarinis et al., 2023). *L. plantarum* H64 and 18S9, instead, were selected for their pro-technological potential, specifically the acidification capacity and proteolysis efficiency (Nionelli et al., 2018a; Perri et al., 2023). A rice/sprouted barley mixture was subjected to gelatinization followed by fermentation. Combining rice and barley flours can influence various aspects of foods, including their physico-chemical properties, nutritional content, and sensory characteristics. Although this combination was never explored for yogurt alternatives, studies on extruded snacks (Gupta et al., 2008), flatbreads (Maiya et al., 2015), and cooked kernel mixtures (Woo et al., 2018), found that the incorporation of barley can *i*) modify the proximal composition providing proteins, dietary fibres, β-glucans (Maiya et al., 2015); *ii*) enhance the radical scavenging activity due to the increase of phenolic compounds (Woo et al., 2018); *iii*) affect viscosity, water binding capacity and swelling power (Gupta et al., 2008; Maiya et al., 2015; Woo et al., 2018).

The preliminary screening of the starter cultures underscored the importance of selecting strains with optimal growth, acidification kinetics, and proteolysis. Overall, all the strains well adapted to the matrix however, independently of the strain used, acidification was found to be extremely high (with pH lower than 3.4), and intense acidification is generally perceived negatively by those who consume plant-based yogurt (Jaeger et al., 2023). Moreover, the low pH values can change LAB metabolism from sugars to amino acids since glutamate-, histidine-, and tyrosine-decarboxylase play an important role in maintaining pH homeostasis and LAB survival (Gänzle, 2015), explaining why a reduction of TFAA content was found. Among all the strains, *L. plantarum* H64 distinguished itself for maintaining stable levels of TFAA, unlike the notable 10–20 % decrease observed with other strains, and preservation of TFAA is crucial as amino acids contribute to flavor enhancement and bioactive properties (Wu, 2021). The slower growth and acidification of *Leuc.* *pseudomesenteroides* DSM20193, characterized by a longer lag phase (Fig. 1), could hinder its application in rapid fermentation processes. In contrast, the balanced metabolic activity of *L. plantarum* H64 makes it an ideal candidate for such gurt production. Moreover, its ability to produce substantial amounts of the functional amino acid GABA was previously demonstrated (Verni et al., 2022), a feature that could enhance the added value of the products developed in this study. Therefore, only *L. plantarum* H64 was selected for further experiments.

The viscosity of the fermented products, initially determined to evaluate the capacity of the strains to synthetize exopolysaccharides, could not be taken into consideration for the starter selection. In fact, at the end of the gelatinization the beverages were liquid. It could be hypothesized that the high amylase activity in sprouted barley might have interfered with the starch gelatinization process. Indeed, amylolytic activity starts increasing few hours after the seed imbibition and further increases during the germination process (Guzmán-Ortiz et al., 2019). Gelatinization, which in the production of plant-based products is one of the fundamental steps necessary to confer a structure resembling that of conventional yogurts (Montemurro et al., 2021; Dhakal et al., 2023), consists in the swelling of starch granules under heat treatment (Zhang et al., 2024). Nevertheless, it was recently demonstrated that the hydrolysis by amylases changes wheat starch structure and prevents it from properly gelatinize (Cheng et al., 2022).

### 4.2 Process optimization

Hence, aimed at demonstrating that sprouted barley was responsible for the lack of gelatinization a new set of products was designed and the production process optimized. Beside the gurts containing sprouted barley (sB-G and sfB-G), two more thesis containing raw barley were added (rB-G and fB-G). To avoid an excessive acidification, fermentation time was decreased from 18 to 8 h, whereas, aiming at deactivating amylolytic enzymes which might have interfered with the gelatinization process, sprouted barley was roasted at 150 °C for 3 h, and the flour used to produce a fifth gurt (sfRB-G). Then, gurts were inoculated with *L. plantarum* H64 and incubated at 30 °C.

By shortening the fermentation time, which was still sufficient to allow the growth of the starters, the pH decrease was not as pronounced as in the starter selection, with the highest value reached in fB-G and sfRB-G. Indeed, compared to sprouted barley flour in sfB-G, raw barley flour provided a lower amount of soluble sugars, which is why the acidification was significantly (P < 0.05) lower in fB-G compared to sfB-G (Table 2). On the other hand, the lower acidification observed in sfRB-G compared to sfB-G, despite containing both sprouted barley flour, is likely due to the thermal treatment aimed at deactivating the amylases. Indeed, as shown in Table 2, at the beginning of fermentation, samples with sprouted barley were the richest in fermentable sugars (glucose, maltose, and sucrose), on the contrary, sfRB-G was the most deficient. It is certainly possible that during the roasting, products of the Maillard reaction were formed between reducing sugars and amino acids (Liu et al., 2022), contributing to the browning of the flour as well as to the reduction of available fermentable sugars and TFAA (Table 2). Moreover, it was also demonstrated that the high amount of glucose upon cereals sprouting accelerates the Maillard reaction (Yıltırak et al., 2022).

Since the aim was to produce a fermented beverage with the consistency of conventional yogurts, viscosity was measured. The results confirmed that the germination of barley was responsible for the unsuccessful gelatinization, since sB-G and sfB-G were liquid compared to rB-G and fB-G, and that the roasting efficiently deactivated the enzymes allowing a proper gelatinization in sfRB-G, comparable to that of commercial dairy yogurt (Vélez-Ruiz, 2008). However, the thermal treatment impacted the nutritional value of the gurt, modifying its amino acid profile. Indeed, although significantly higher (P < 0.05) than that found in fB-G, free amino acids content, including that of GABA, was from 9 to 69 % lower in sfRB-G compared to sfB-G. GABA has been shown to have several physiological functions including regulating circulatory system and reducing the risk of cardiovascular diseases by modulating cholesterol levels (Hou et al., 2024). It also has been shown to reduce the risks of type I diabetes, improving insulin response; and several *in vivo* studies have proven that the consumption of GABA-rich foods can reduce blood pressure and cholesterol and may play a role in the management of depression and anxiety (Hou et al., 2024). All fermented gurts produced in this study contained higher GABA content (up to 67 mg/kg) compared to the respective controls, and higher than other values found in the literature for yogurt alternatives (Demarinis et al., 2023). However, a recent study focusing on the optimization of GABA in a yogurt alternative obtained with sprouted soybean, contained up to 2 g/L (Zhang et al., 2023). It should be pointed out that the process conditions where highly different from our study. For instance, soybean was used at a ratio 1:2 with water, whereas in the conditions optimized in this study, sprouted barley constituted only 5 % of the gurt, hence it was more diluted. Moreover, to increase GABA synthesis during fermentation with *Lb.* *brevis* NPS-QW 145, the mixture was added of monosodium glutamate, substrate for the reaction (Zhang et al., 2023). Nevertheless, despite the lower yield compared to that found by Zhang et al. (2023), the values reached in this study might be sufficient to obtain a functional product. It was reported that the consumption of 10 mg of GABA per day for 12 weeks is effective in controlling blood pressure in mildly hypertensive patients (Inoue et al., 2003). Hence, it could be hypothesized that the consumption of 150 g of sfB-G per day could have the same effect. On the contrary, for sfRB-G to have the same effect, it would be necessary a consumption of 500 g, thus highlighting the intricate relationship among processing parameters and nutritional, rheological, and sensory aspects. In fact, while on one side, the thermal treatment on sprouted barley was necessary to achieve the desired consistency, it also led to amino acids depletion and the consequent formation of VOC like pyrazines. Indeed, while 1-Octen-3-ol, 3-methylbutanal, 2-methylbutanal, hexanal, 2-hexenal, 2-heptenal, 2-nonenal, and decanal were identified as key flavor compounds in several barley varieties (Cramer et al., 2005), pyrazines as are not. Pyrazines, volatile compounds that share monocyclic aromatic hydrocarbon with a counterpoint nitrogen atom, quickly form when processing temperatures exceed 120 °C and are responsible for the typical roasting flavor found in bread, cocoa, coffee, and roasted nuts (Zhao et al., 2021) thus explaining the toasted notes perceived during the sensory analysis of sfRB-G. The VOC analysis also revealed significant diversity between raw and sprouted barley-based gurts, not only in the relative abundance of the identified compounds, but also in terms of the type of compound. The separation in the PCA (Fig. 4) was driven by alcohols, furans, and aldehydes among which 2-pentylfuran, the most representative in sprouted barley, is known for the butter, green bean, and floral flavors (Perri et al., 2021) thus emphasizing the contribution of germination to the aroma profile. Overall, all the sensory attributes differentiating the gurts for color, aroma, taste, and texture (Fig. 6) are in line with the processing performed and the biochemical characterization (Fig. 5, Table 2).

The stability of the key biochemical and microbiological parameters during refrigerated storage was also assessed. The gradual pH decrease, the rise in TTA and amino acids content (especially in sfRB-G) during the first two weeks reflected the continued microbial activity of the starters, which remained vital and above 9 log cfu/g for the entire storage and are consistent with prior observations in similar gurts (Demarinis et al., 2023, 2024).

## 5 Conclusions

To conclude, plant-based gurts made with cereal flours, notably barley, emerge as a sustainable and health-promoting substitute to traditional dairy yogurts. The incorporation of germinated grains, combined with fermentation by lactic acid bacteria, offers a pathway to develop nutrient-rich and environmentally friendly products. Notwithstanding, the equilibrium between favorable and unfavorable attributes in plant-based formulations remains a critical area for innovation. The challenge provided by the liquid texture of sprouted barley-based alternatives can be overcome by some adjustments in the production process (e.g., the thermal treatment) to meet consumers expectations for creamy and spoonable products with both nutritional and sensory profiles superior to those obtained with raw barley. However, if by weighing in the balance between nutritional and rheological features, aspects like the content of functional compounds (e.g., GABA or TFAA) hold the highest weight for both consumers and industry, the addition of structuring agents should be considered. Still, the interplay between matrix composition and bioprocessing dynamics offers a rich landscape for innovation in plant-based dairy substitutes.

## CRediT authorship contribution statement

**Mario Caponio:** Investigation, Formal analysis, Writing – original draft. **Michela Verni:** Conceptualization, Validation, Formal analysis, Writing – review & editing, Visualization, Supervision. **Ali Zein Alabiden Tlais:** Investigation, Writing – review & editing. **Edoardo Longo:** Investigation. **Erica Pontonio:** Supervision. **Raffaella Di Cagno:** Resources, Writing – review & editing. **Carlo Giuseppe Rizzello:** Conceptualization, Resources, Writing – review & editing, Supervision, Funding acquisition.

## Ethical statement

The sensory analysis was approved by the local health board of Lazio region (n. 0998/2024). Informed consent of all participants was obtained prior to participating in sensory analysis. Appropriate protocols for protecting the rights and privacy of all participants were utilized during the execution of the sensory analysis.

## Funding

This research did not receive any specific grant from funding agencies in the public, commercial, or not-for-profit sectors.

## Declaration of competing interest

The authors declare that they have no known competing financial interests or personal relationships that could have appeared to influence the work reported in this paper.

## Data Availability

Data will be made available on request.
